# The Attenuated *Brucella abortus* Strain 19 Invades, Persists in, and Activates Human Dendritic Cells, and Induces the Secretion of IL-12p70 but Not IL-23

**DOI:** 10.1371/journal.pone.0065934

**Published:** 2013-06-21

**Authors:** Mario Weinhold, Martin Eisenblätter, Edith Jasny, Michael Fehlings, Antje Finke, Hermine Gayum, Ursula Rüschendorf, Pablo Renner Viveros, Verena Moos, Kristina Allers, Thomas Schneider, Ulrich E. Schaible, Ralf R. Schumann, Martin E. Mielke, Ralf Ignatius

**Affiliations:** 1 Institute of Microbiology and Hygiene, Department of Infection Immunology, Charité – Universitätsmedizin Berlin, Berlin, Germany; 2 Robert Koch-Institute, Berlin, Germany; 3 Institute of Tropical Medicine and International Health, Charité – Universitätsmedizin Berlin, Berlin, Germany; 4 Medical Clinic I, Charité – Universitätsmedizin Berlin, Campus Benjamin Franklin, Berlin, Germany; 5 Research Center Borstel, Department of Molecular Infection Research, Borstel, Germany; National Council of Sciences (CONICET), Argentina

## Abstract

**Background:**

Bacterial vectors have been proposed as novel vaccine strategies to induce strong cellular immunity. Attenuated strains of *Brucella abortus* comprise promising vector candidates since they have the potential to induce strong CD4^+^ and CD8^+^ T-cell mediated immune responses in the absence of excessive inflammation as observed with other Gram-negative bacteria. However, some *Brucella* strains interfere with the maturation of dendritic cells (DCs), which is essential for antigen-specific T-cell priming. In the present study, we investigated the interaction of human monocyte-derived DCs with the smooth attenuated *B. abortus* strain (S) 19, which has previously been employed successfully to vaccinate cattle.

**Methodology/Principal findings:**

We first looked into the potential of S19 to hamper the cytokine-induced maturation of DCs; however, infected cells expressed CD25, CD40, CD80, and CD86 to a comparable extent as uninfected, cytokine-matured DCs. Furthermore, S19 activated DCs in the absence of exogeneous stimuli, enhanced the expression of HLA-ABC and HLA-DR, and was able to persist intracellularly without causing cytotoxicity. Thus, DCs provide a cellular niche for persisting brucellae *in vivo* as a permanent source of antigen. S19-infected DCs produced IL-12/23p40, IL-12p70, and IL-10, but not IL-23. While heat-killed bacteria also activated DCs, soluble mediators were not involved in S19-induced activation of human DCs. HEK 293 transfectants revealed cellular activation by S19 primarily through engagement of Toll-like receptor (TLR)2.

**Conclusions/Significance:**

Thus, as an immunological prerequisite for vaccine efficacy, *B. abortus* S19 potently infects and potently activates (most likely via TLR2) human DCs to produce Th1-promoting cytokines.

## Introduction

Novel vaccine strategies for the induction of cellular immune responses are based on the use of suitable microbial shuttles, which include the genetic information encoding immunogenic epitopes of the targeted pathogen. Besides various viral vectors, such as adenoviruses or poxviruses [1], certain bacterial vaccine strains have been engineered successfully. For instance, strains of the attenuated *Salmonella enterica* serovar Typhi with antigens derived from pathogens, such as *Helicobacter pylori*, *Plasmodium falciparum*, or Hepatitis B virus, have already been tested in volunteers. Thus far, only a minority of the vaccines, however, was successful in inducing substantial cellular or humoral immune responses [2].

*Brucella abortus* is a Gram-negative alpha-proteobacterium and the cause of bovine brucellosis. Since the lipopolysaccharide (LPS) of brucellae is less pyrogenic than enterobacterial LPS, brucellae may be advantageous as vaccine vectors [3]. Both Th1 CD4^+^ and CD8^+^ T cell subsets are activated during the course of experimental infection [4], [5]. Antigenic preparations of brucellae, such as heat-inactivated bacteria or DNA, have been used as adjuvants for the induction of systemic and mucosal Th1 immune responses in mice [6]–[10] and non-human primates [11].

Two attenuated *B. abortus* strains have been developed to control bovine brucellosis, i.e., the smooth strain (S)19 and the rough one, RB51[12]. Both strains induce potent cellular immune responses in mice [5], [13]–[15], and have been used to develop live or replication-incompetent vectors for exogenous antigens [16], [17]. Recombinant strain RB51 expressing the antigens of *Neospora caninum*, MIC1 and GRA6, for instance, induces protective immune responses against a lethal murine *N. caninum* infection [18]. Notably, rough brucellae have been shown to induce higher amounts of various cytokines and chemokines in human monocytes in vitro than smooth strains [19]. Therefore, recombinant vectors differing in phenotypical characteristics, such as their type of LPS, may also differ with respect to the immune responses induced in vivo.

Although clinical manifestations of accidental infections with S19 in humans were mild [20], and a related strain, S19-BA, has been used in the former USSR as a vaccine in humans [21], the safety profile of the present S19 (without further attenuation) is not adequate for its use in humans [21].

Essential for the use of brucellae as vectors for the induction of cellular immune responses is their interaction with antigen-presenting cells. Activation of monocytes [22], [23], macrophages [23], as well as neutrophils [24] by brucellae has been reported. Notably, different *Brucella* spp. including a GFP-expressing mutant of the virulent *B. abortus* strain 2308 can also readily infect dendritic cells (DCs) [25]. As DCs, which are matured as a consequence of cellular activation, are the most potent antigen-presenting cells for the priming of naïve T cells and thereby induction of cellular immune responses, this cell type is of particular interest in vaccine immunology [26]. In mice, DCs are the major source of IL-12 when heat-killed brucellae are used as an adjuvant [9]. Zwerdling et al. [27] have demonstrated that the virulent smooth *B. abortus* strain, 2308, also activates human DCs leading to the secretion of cytokines, e.g., IL-12p40 and IL-10, and that *Brucella*-derived lipoproteins represent the inflammatory principle of brucellae. However, the study did not address whether the bacteria themselves were sufficient for the induction of DC-activation or whether an autocrine cytokine-mediated effect further matured the cells. Furthermore, it has not been further differentiated whether the IL-12p40 measured was paired to either p35 or p19 thereby composing IL-12p70 or IL-23, respectively, which is of relevance for the T-cell response induced [28].

In contrast to the potential of brucellae to activate DCs, *B. suis* has been reported to prevent human DC-maturation due to the failure of the cells to produce TNF-α [29]. Likewise, *B. abortus* affects the maturation of murine DCs, and this effect has been attributed to the *Brucella* protein, Btb1, which interferes with the Toll-like receptor (TLR)2 signaling pathway [30].

As the strong T-cell stimulating potential of the smooth attenuated *B. abortus* S19 in vivo has been documented before [13], [15], we were interested in the possible underlying mechanisms. In the present study, we investigated the effects of S19 on the cytokine-induced maturation of monocyte-derived DCs as well as on immature DCs. We show that S19 does not interfere with but modulates the cytokine-induced DC-maturation. Furthermore, S19 potently activates immature DCs most likely through TLR2/TLR6, persists intracellularly without causing excessive cytotoxicity, and induces the secretion of IL-12p70 identifying it as a potential source for the future development of a bacterial vector for vaccine antigens requiring Th1 responses.

## Materials and Methods

### Bacteria

*B*. *abortus* S19 was kept virulent by mouse passages. Cultures obtained from spleen homogenates were grown in tryptic soy broth, harvested in log phase, dispensed in 0.5 ml aliquots, and frozen at –70°C until needed [15]. For experiments using heat-inactivated S19, bacteria were inactivated by incubation at 70°C for 30 min or 20 h. *B abortus* 2308 was grown similarly and inactivated by incubation at 70°C for 20 h. Inactivation was verified by the lack of bacterial growth in tryptic soy broth. All procedures involving live *B. abortus* 2308 were performed under BSL-3 conditions. Inactivated bacteria were stored at −60 to −80°C until use.

### Generation of human DCs

Buffy coats (German Red Cross, Berlin) or peripheral blood from healthy volunteers (obtained from lab members who gave their verbal informed consent, documented in lab notebooks, and approved by the ethics committee of the Charité, 119 EA1/316/12) were used to isolate PBMCs. Monocyte-derived DCs were generated from magnetically separated CD14^+^ monocytes (Miltenyi Biotec, Bergisch-Gladbach, Germany) as previously described [31]. Briefly, monocytes were cultured at 3×10^6^ cells/well in 6 well cell culture dishes (Nunc, Roskilde, Denmark; or TPP, Trasadingen, Switzerland) for 6 days in RPMI 1640, supplemented with 2 mM L-glutamine, 50 µM 2-mercaptoethanol, 10 mM HEPES (all GIBCO, Invitrogen, Karlsruhe, Germany), recombinant human GM-CSF (1000 U/ml, sargramostim, Leukine®, Berlex, Richmond, CA), recombinant human IL-4 (100 U/ml, R&D Systems, Wiesbaden-Nordenstadt, Germany), and 10% heat-inactivated FCS (Biochrom, Berlin, Germany), adding fresh medium and cytokines every other day. In some experiments, cells were matured by recombinant human IL-6, tumor necrosis factor alpha (TNF-α), IL-1β (all 10 ng/ml, R&D Systems) and 10 µM PGE_2_ (Sigma, Taufkirchen, Germany) [31], [32].

### Infection of DCs with *B. abortus* S19

DCs were incubated with *B. abortus* S19 at MOI 20 in antibiotic-free medium for 60 min at 37°C and 5% CO_2_. To remove extracellular bacteria, cell cultures were washed five times with medium followed by incubation in the presence of gentamicin (100 µg/ml; Biochrom) for 90 min at 37°C and 5% CO_2_. Cells were washed another three times in medium, applied to the individual experiments, and gentamicin (10 µg/ml) was added to the cultures to sustain the control of the bacteria.

To determine numbers of intracellular bacteria, cells were washed to remove the gentamicin, counted, and lysed in 0.2% Triton-X-100/PBS for 30 min at 37C° and 5% CO_2_ [33]. Suspensions were spread in serial dilutions on tryptic soy agar or Columbia agar, and colonies were counted after 5 d incubation at 37°C and 5% CO_2_.

### Flow cytometry

The phenotype of human DCs was monitored by flow cytometry with PE- or FITC labeled anti-human mAbs to the following surface molecules: HLA-DR, CD14, CD25, CD80, CD86 (all BD Pharmingen), CD83 (Caltag Laboratories, Hamburg, Germany), HLA-ABC (Dako; BIOZOL Diagnostica, Eching, Germany), or the appropriate isotype controls, as previously described [31]. For detection of intracellular antigens, cells were fixed for 30 min in 4% paraformaldehyde/PBS (w/v), permeabilized by washing twice in 0.5% saponin (Sigma), and subsequently stained with rabbit anti-*Brucella*-LPS serum (BD) for 60 min in 0.5% saponin, followed by incubation with a secondary FITC-conjugated goat anti-rabbit IgG mAb (Jackson) for another 60 min in 0.5% saponin. All cell populations were fixed with 10% formaldehyde/PBS (v/v) before analysis on a FACSCalibur® cytometer with CELLQuest®Pro software (BD Biosciences).

### Analysis of cytokine secretion by DCs

48 h cell-free supernatants of DC cultures were harvested and stored at –80°C until analysis by sandwich ELISAs for IL-12/23p40, IL-12p70, IL-10, TNF-α (all U-CyTech, Utrecht, The Netherlands), or IL-23 (eBioscience).

### Analysis of the involvement of TLRs in the cellular recognition of *B. abortus* S19 or *B. abortus* 2308

To investigate the involvement of different TLRs in immune cell stimulation by *B. abortus* S19 and strain 2308, the human embryonal kidney cell line HEK 293 was employed as described previously [34]. In brief, cells were cultured in DMEM medium (Gibco) including sodium pyruvate supplemented with 10% fetal bovine serum, 10 kU/ml penicillin, 10 g/ml streptomycin, and 200 mM L-glutamine. For stimulation experiments, cells were cultured at a density of 1×10^5^ cells/well in 12-well tissue culture plates over night. Transfection with expression plasmids encoding ß-galactosidase (0.04 µg) and the ELAM NF-κB luciferase reporter plasmid (0.12 µg, both kindly provided by C. J. Kirschning, Essen, Germany) for 24 h was performed employing 0.5 µl/well Fugene® (Roche, Mannheim, Germany). Cells were additionally transfected with hTLR-2, and in addition hTLR1 or TLR6, or with hTLR4 and MD-2 as indicated. Cells were stimulated for 20 h with inactivated brucellae, the lipopeptide Pam2Cys (InvivoGen, San Diego, CA, USA), or LPS (from *S. enterica* Minnesota Re595, Sigma; or from *Escherichia coli* EH100, Alexis Biochemicals, Enzo Life Sciences, Lörrach, Germany), as indicated in 0.5 ml DMEM medium without FCS, followed by lysis and measurement of ß-galactosidase and luciferase activity employing a kit based on chemiluminescence (Roche, Mannheim, Germany). Chemiluminescence values were normalized for transfection efficacy indicated by ß-gal values and given as relative light units (RLU). Experiments were performed in triplicates and repeated once. In addition, IL-8 concentrations in supernatant were determined by using a paired IL-8 mAb sandwich ELISA (BD Biosciences).

### Statistics

Statistical significance of differences was determined by the Wilcoxon matched-pairs signed rank test (data expressed as median with interquartile range) or the paired Student's *t* test (data expressed as mean ± standard deviation) using GraphPad Prism version 6.0a for Mac OS X. Differences were considered statistically significant for p<0.05.

## Results

### *B. abortus* S19 does not interfere with the maturation of human DCs but alters their cytokine-secretion pattern

**Figure 1:**
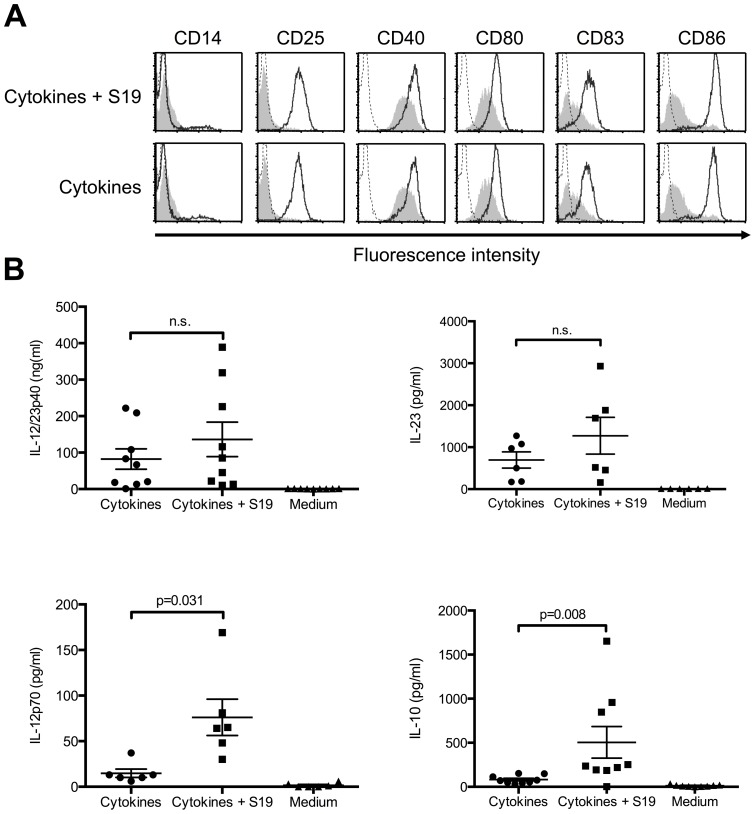
*B.* *abortus* S19 does not interfere with the cytokine-induced maturation of immature human DCs. Monocyte-derived immature DCs were infected with *B. abortus* S19 (MOI, 20) for 1 h, the bacteria were washed out, and the cells incubated in the presence of pro-inflammatory cytokines (TNF-α, IL-1β, IL-6, PGE_2_). Uninfected control cells were incubated alongside in the presence or absence of cytokines. (A) After 48 h, the phenotype of the cells was determined by flow cytometry (bold lines, stimulated cells; grey areas, unstimulated cells; dotted lines, isotype controls). One representative of at least six independent experiments is shown. (B) Supernatants were analyzed for the presence of IL-12/23p40, IL-12p70, IL-23, and IL-10 (data expressed as median with interquartile range; n.s., not significant).

It has recently been shown that *B. suis* and *B. abortus* may prevent the maturation process of DCs [29], [30]. To investigate whether *B. abortus* S19 similarly inhibits DC-maturation, we infected immature human DCs (MOI, 20) for 1 h, washed the cells, added antibiotics, and cultured the cells in the presence of pro-inflammatory cytokines, which lead to the maturation of DCs and may as well be produced in the course of human brucellosis [31], [32]. Uninfected control cells also were incubated in the presence of cytokines or kept immature by culturing in the absence of additional cytokines. After 48 h, we harvested the cells and monitored the expression of DC maturation markers by flow cytometry. Upon co-culture in the presence of cytokines, both infected and uninfected DCs showed up-regulated surface expression of markers typically expressed by mature DCs whereas the uninfected control cells cultured in the absence of cytokines were negative for CD25 and expressed considerably less CD40, CD80, CD83, and CD86 than cytokine-matured cells (Figure 1A). CD25, CD80, CD83, and CD86 were significantly higher expressed by cytokine-incubated, uninfected or infected DCs than by control cells incubated in medium alone while infected and uninfected, cytokine-incubated DCs did not differ regarding the expression of these molecules (Figure S1). The viability of infected and uninfected DCs was comparable (infected, 76.86% +/− 19.09%; uninfected, 84.86% +/− 11.61%; n = 7; p = 0.210). Thus, *B. abortus* S19 does not interfere with the differentiation of human DCs upon maturation by pro-inflammatory cytokines nor does it show cytotoxic effects.

Since mature DCs produce cytokines involved in T-cell activation and differentiation, we were interested whether the infection with S19 might inhibit the cytokine production or alter the cytokine profile of activated DCs. We therefore analyzed the concentrations of IL-12/23p40, IL-23, IL-12p70, and IL-10 in the supernatants collected 48 h after adding the cytokines to freshly infected or uninfected DCs. We detected similar concentrations of IL-12/23p40 and IL-23 in supernatants from infected and uninfected cells (Figure 1B). However, secretion of both the Th1-driving cytokine, IL-12p70, and the anti-inflammatory cytokine, IL-10, was significantly enhanced in cytokine-matured and infected DCs as compared to uninfected cells. Uninfected, immature control cells kept in medium did not produce substantial amounts of any of these cytokines. Therefore, infection of DCs with *B. abortus* S19 does not inhibit but modifies the maturation-induced secretion of cytokines, i.e., it significantly enhances the secretion of IL-12p70 and IL-10.

### *B. abortus* S19 activates immature human DCs

To further analyze the increased production of IL-12p70 and IL-10 by infected DCs (Figure 1B), we infected immature DCs with *B. abortus* S19 in the absence of additional cytokines and analyzed the phenotype of the cells 48 h later. Immature cells were kept uninfected as control.

**Figure 2:**
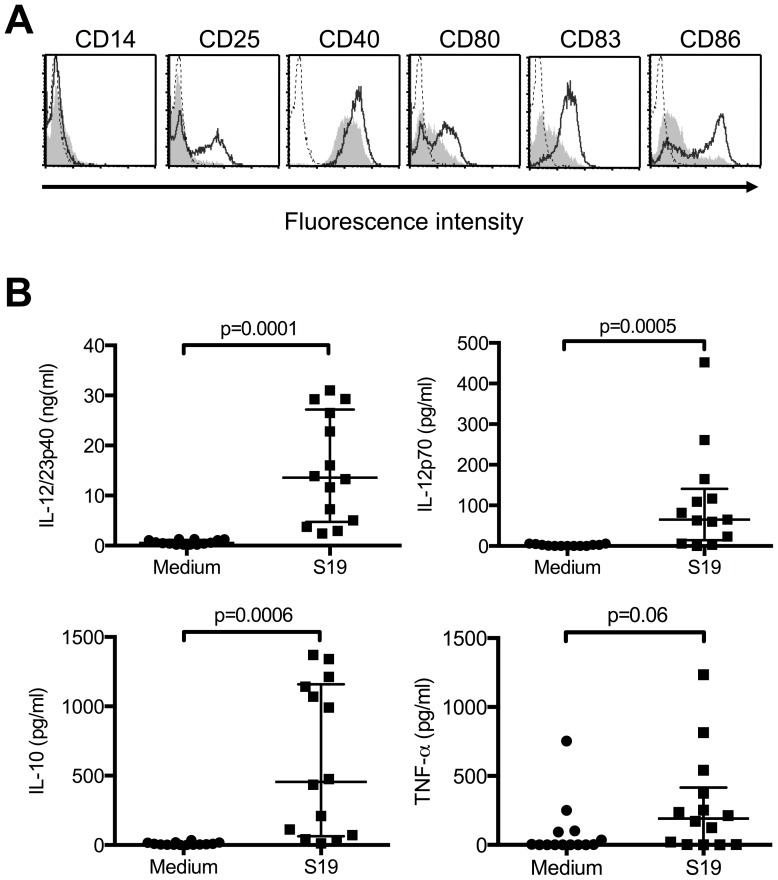
Human immature DCs are activated by *B. abortus *S19. Monocyte-derived immature DCs were infected with *B. abortus* S19 (MOI, 20) for 1 h, the bacteria were washed out, and the cells were incubated for another 48 h. Control cells were left uninfected. (A) After 48 h, the phenotype of the cells was determined by flow cytometry (bold lines, infected cells; grey areas, uninfected cells; dotted lines, isotype controls). One representative of at least six independent experiments is shown. (B) Supernatants were analyzed for the presence of IL-12/23p40, IL-12p70, IL-10, and TNF-α. Data expressed as median with interquartile range.

Infected DCs expressed CD25, CD40, CD80, and CD86 significantly higher on their surface than control cells (Figure 2A, Figure S2). Although statistically not significant, there also was a strong trend (p = 0.06) for a higher expression of CD83 following exposure of the cells to *B. abortus* S19. Notably, infected and uninfected DCs did not differ regarding their viability (infected, 74% +/− 28.23%; uninfected, 70.71% +/− 30.18%; n = 7; p = 0.898). Correlating with the phenotypic pattern of activated cells, infected DCs secreted substantial amounts of IL-12/IL-23p40, which was partially produced as IL-12p70 (Figure 2B), whereas IL-23 was not detected in supernatants from infected nor uninfected DCs (data not shown). Furthermore, infected DCs but not uninfected control cells also produced IL-10 and TNF-α. Therefore, S19 potently activates human DCs to produce mainly Th1-promoting cytokines, which is in line with the strong T-cell inducing effect of *B. abortus* S19 in vivo [13], [15].

### *B. abortus* S19 induces higher expression of HLA-ABC and HLA-DR on human DCs in the absence of pro-inflammatory cytokines

**Figure 3:**
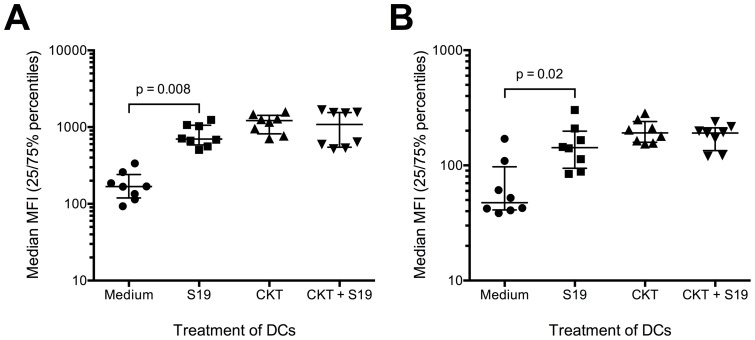
Increased MHC expression by strain S19-infected DCs. Immature DCs were infected with *B. abortus* S19 at MOI 20, the bacteria were washed out, antibiotics were added, and the cells were then incubated in the presence or absence or pro-inflammatory cytokines. After 42 h, the cellular expression of HLA-ABC (A) or HLA-DR (B) was determined by flow cytometry. Medians of the MFIs as well as the 25% and 75% percentiles of the MFIs for cells from eight donors (MFIs for isotype controls did not exceed 3.5). * p<0.05 compared to untreated DCs (Wilcoxon matched-pairs signed rank test).

Since high expression of MHC molecules is required for efficient antigen presentation by DCs, we next determined whether *B. abortus* S19 alters the expression of these molecules on monocyte-derived DCs. Immature DCs were infected with S19 while control cells were kept uninfected. The bacteria were washed out and cells were incubated in the presence or absence of pro-inflammatory cytokines as before. Two days later, HLA-ABC and HLA-DR expression was analyzed by flow cytometry. Infected cells incubated in the absence of cytokines expressed both HLA-ABC and HLA-DR at higher levels than uninfected controls cells (Figure 3). In contrast, there was no difference between infected and uninfected cells incubated in the presence of cytokines regarding HLA-ABC (p = 0.6) or HLA-DR (p = 0.3). Thus, infection with S19 increases MHC expression on human DCs in the absence of cytokine stimulation while it does not further enhance the expression of these molecules on cytokine-matured cells.

### Pro-inflammatory cytokines limit the persistence of *B. abortus* S19 in DCs

**Figure 4:**
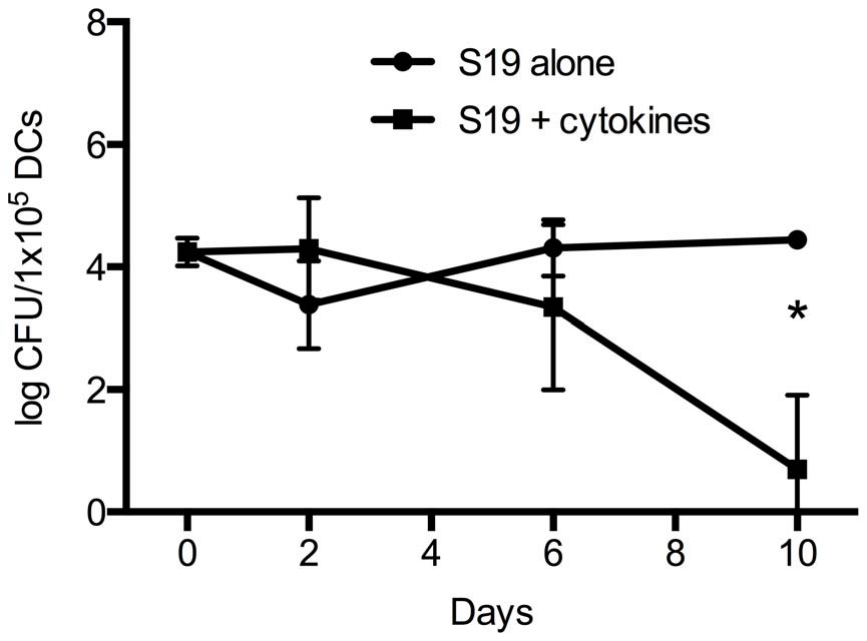
Prolonged intracellular survival of *B.* *abortus* S19 in unstimulated DCs compared with cytokine-matured DCs. Immature DCs were infected with *B. abortus* S19 at MOI 20, the bacteria were washed out, antibiotics were added, and the cells were then incubated in the presence or absence or pro-inflammatory cytokines. Cells were lysed at day 0 to determine the initial number of intracellular bacteria, and on days 2, 6, and 10 to ascertain the intracellular bacterial numbers over time. CFUs differed significantly at d10 (*, p<0.05).

Since *B. abortus* S19 invaded DCs without apparent cytotoxic effects, we were interested in whether the bacteria may persist in DCs and in monitoring the cellular differentiation state over time. We therefore cultured infected cells in the presence or absence of cytokines, harvested cells at days 0, 2, 6, and 10, and determined their maturation state by flow cytometry as well as the intracellular CFUs after lysis of the cells. *B. abortus* S19 survived in cytokine-incubated DCs for at least 6 days. At day 10, we detected viable bacteria at low CFU in only one out of three experiments. In contrast, in the absence of pro-inflammatory cytokines, bacteria persisted over the entire observation period, and at d 10, significantly more bacteria could be cultured from these DCs than from cells incubated in the presence of cytokines (Figure 4; p = 0.034, paired Student's *t* test). The morphology of the bacterial colonies was smooth in all experiments, i.e., we did not observe any phenotypic change of S19 towards a rough colony type. Thus, when *B. abortus* S19 invades DCs that are cultured in the presence of pro-inflammatory cytokines, numbers of intracellular bacteria decline over time. In contrast, after infection of immature DCs, the bacteria can persist for at least 10 days despite concurrent cellular activation, and such cells might provide a cellular niche for the bacterial persistence and a source of T-cell stimulating brucella antigens *in vivo*.

**Figure 5:**
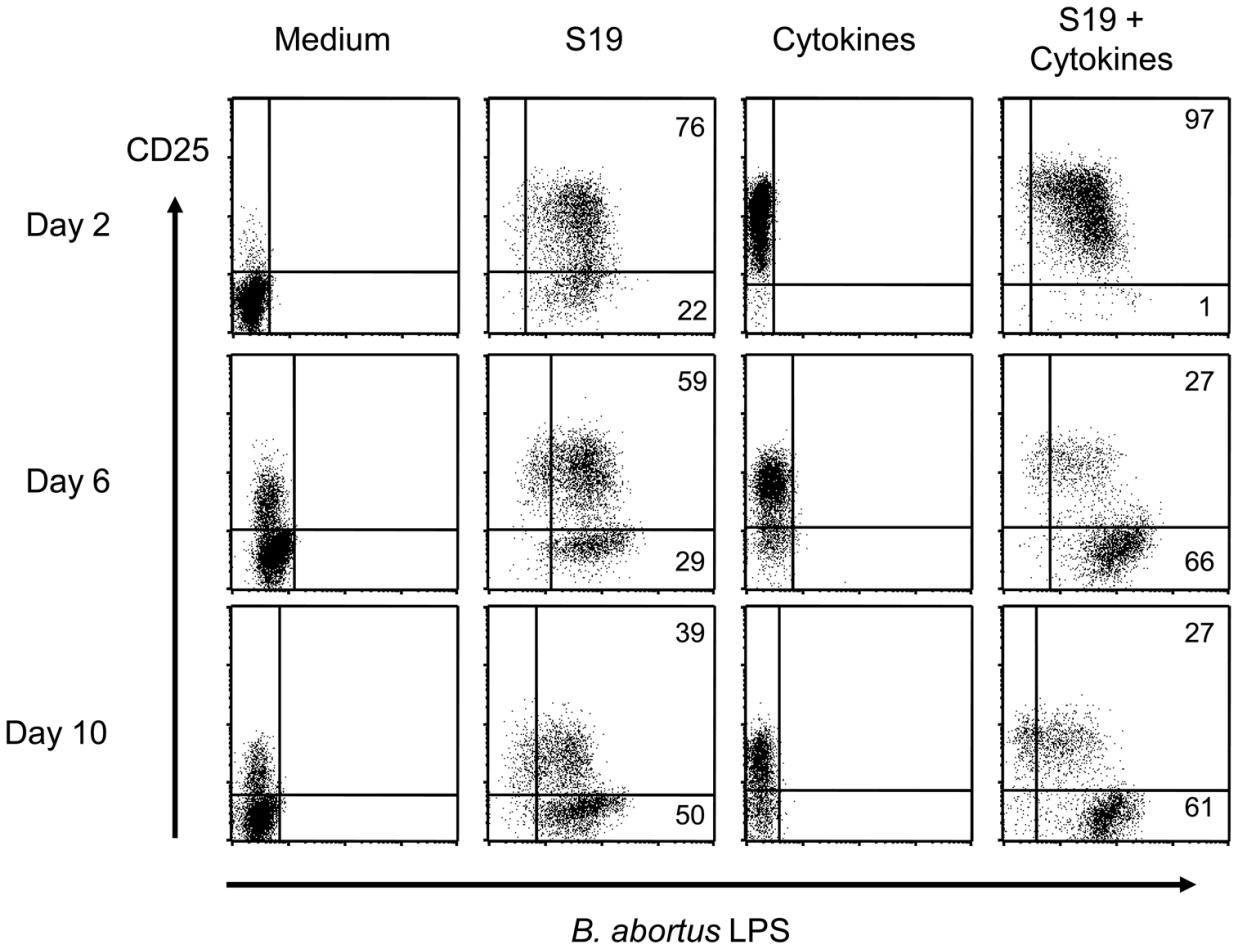
*B.* *abortus* strain S19 persists in less mature cells in both medium-incubated and cytokine-matured DCs. Immature DCs were infected with *B. abortus* S19 at MOI 20, the bacteria were washed out, antibiotics were added, and the cells were incubated in the presence or absence or pro-inflammatory cytokines. At days 2, 6, and 10, cells was harvested and analyzed by flow cytometry for their extracellular expression of CD25 and the intracellular content of Brucella-LPS. One representative experiment of three independent experiments is shown.

To identify Brucella-positive cells within DC populations, we applied intracellular staining for the detection of brucella-positive cells by using antibodies against Brucella-LPS in combination with mAbs binding to surface molecules typically expressed by mature DCs. Simultaneous staining of infected cells for both maturation markers and Brucella LPS revealed that the majority of CD25-positive DCs at day 2 also contained Brucella (or LPS thereof). At later time points, however, LPS-positive DCs became negative for CD25 in a time-dependent manner. This was particularly pronounced in DCs incubated in the presence of cytokines where most of the LPS-positive cells did not express CD25 at days 6 and 10 post infection (Figure 5). Therefore, *B. abortus* S19 might survive better in less mature than in fully mature DCs. Alternatively, infected cells might down-regulate certain DC maturation markers.

### Brucella-induced DC-activation is due to the direct interaction of the cells with the bacteria and not due to soluble mediators

**Figure 6:**
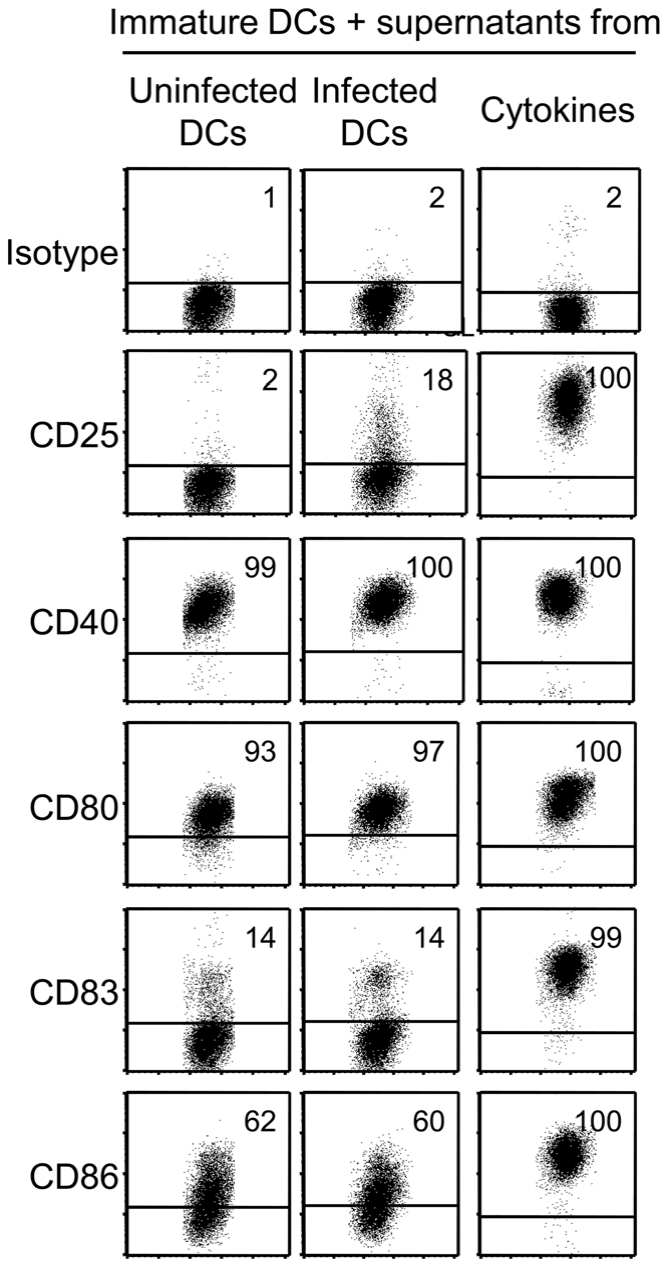
Failure of supernatants from *B.* *abortus* S19-infected DCs to transfer the DC-activating potential to immature, uninfected DCs. Immature cells were infected with *B. abortus* S19 (MOI, 20) or left uninfected and cultured for 48 h. Supernatants were harvested and transferred (50% vol/vol) to uninfected, immature DCs, and the phenotype of the cells was determined after 48 h. The expression of typical DC-maturation markers vs. size (FSC) is shown. One representative experiment of three independent experiments with comparable results is shown.

To investigate whether Brucella-infected cells may secrete cytokines that induce a bystander-maturation of uninfected cells, or alternatively, that brucellae produce a soluble DC-maturing factor, we collected supernatants from DCs 48 h post infection, incubated immature DCs for 48 h in the presence of these supernatants or supernatants from uninfected cells, and determined the phenotype by flow cytometry. Further controls included uninfected, immature DCs incubated in the presence of pro-inflammatory cytokines and cells kept in medium with GM-CSF and IL-4 at an immature state. DCs exposed to supernatants from S19-infected DCs and cells exposed to supernatants from uninfected DCs did not differ considerably regarding their phenotype, i.e., both populations expressed CD25, CD83, and CD86 at substantially lower levels than DCs incubated in the presence of cytokines (Figure 6). These data suggested that the amounts of TNF-α (and possibly other pro-inflammatory cytokines, such as IL-1β or IL-6) produced by Brucella-infected DCs were not sufficient for the induction of phenotypic maturation of accompanying, uninfected cells. To verify this finding, we incubated immature DCs in medium supplemented with concentrations of recombinant TNF-α comparable to those found in supernatants of infected cells (200 pg/ml). However, this treatment neither led to phenotypically matured DCs (data not shown).

**Figure 7:**
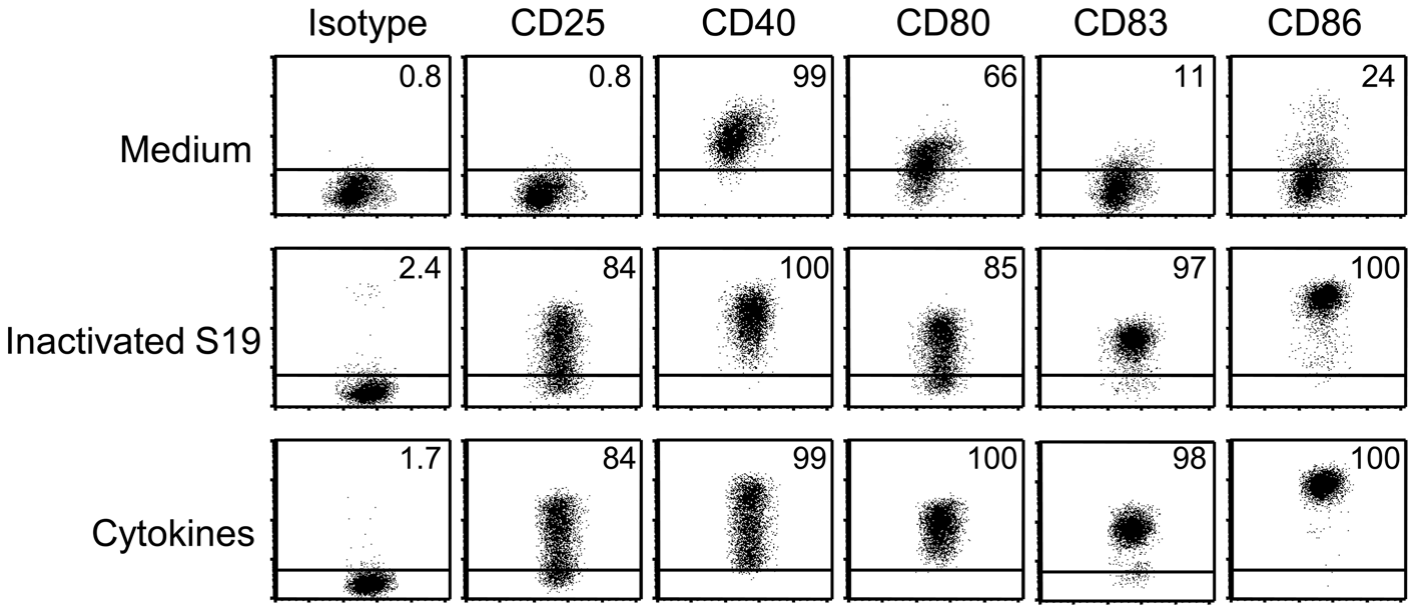
Inactivated *B.* *abortus* S19 activates monocyte-derived DCs. Immature DCs were incubated in the presence of heat-inactivated *B. abortus* S19 (equivalent to MOI 10) or kept in GM-CSF and IL-4 as immature cells. After 48 h, the phenotype of the cells was characterized by flow cytometry. One representative experiment of six independent experiments with comparable results is shown.

To further analyze the underlying mechanism of *B. abortus* S19-induced DC-activation, we incubated immature DCs with heat-inactivated S19. Phagocytosed bacteria were readily detected using the antibody to brucella LPS 4 h later (data not shown). At 48 h, *Brucella*-treated cells expressed a matured phenotype, i.e., most of the cells expressed CD25 and CD83, and the entire population stained positive for CD40, CD80, and CD86 (Figure 7). CD25, CD80, CD83, and CD86 were significantly higher expressed by *Brucella*-treated DCs than by control cells (Figure S3). These data demonstrate that the activation of immature human DCs by *B. abortus* S19 most likely depends on the interaction of cellular pattern-recognition receptors with bacterial antigens.

### *B. abortus* S19 is mainly recognized by TLR2

In order to identify the pattern-recognition-receptor pathway involved in DC-activation by *B. abortus* S19 observed before, we studied TLRs as putative receptors. Data regarding the recognition of brucellae through TLRs are ambiguous and both TLR2 and TLR4 have been reported to be involved depending on the bacterial species and strains as well as on the experimental conditions used [35]. In order to extend these results to S19, we employed a TLR overexpression system based on the human embryonal kidney cell line HEK293 that is usually unresponsive to bacterial stimuli [34], and analyzed TLR4/MD2, TLR2/TLR1, and TLR2/TLR6 heterodimers. Although *B. abortus* DNA is recognized by TLR9, and IL-12p40 secretion of murine splenic DCs or macrophages in vitro depends on TLR9 [36], [37], we did not include TLR9 in our experimental setup since it is expressed by murine but not by human myeloid antigen-presenting cells including DCs [38], [39].

**Figure 8:**
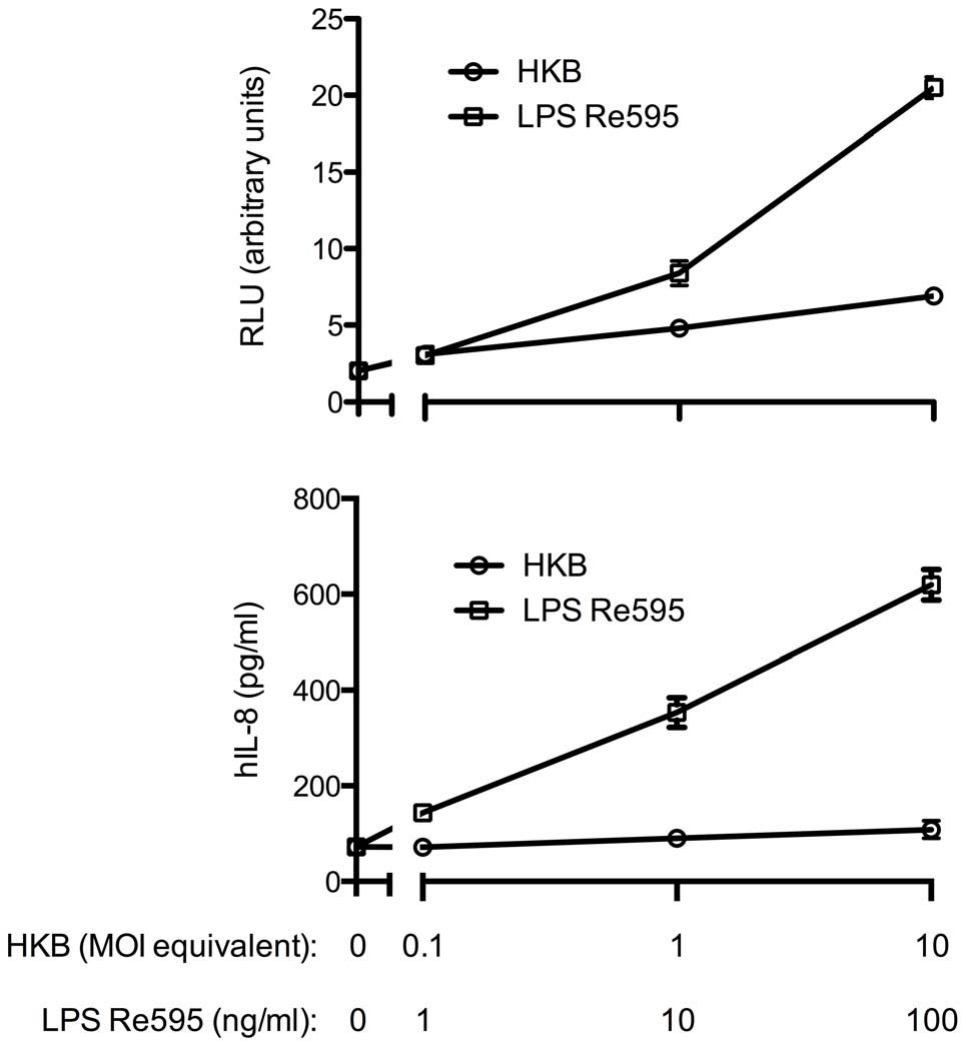
*B.* *abortus* S19 largely fails to induce NF-κB activation in HEK293 cells overexpressing TLR4 and MD-2. HEK293 cells were transiently transfected with human TLR4 and MD-2. After stimulation with LPS or heat-inactivated *B. abortus* S19, NF-κB activation was assessed by a reporter gene assay (A), and IL-8 release by ELISA (B). Cells not transfected with TLR4 and MD-2 did not exhibit NF-κB-activation (not shown). Measurements were performed in triplicates, shown are mean values ± SD. One representative experiment out of two is shown.

**Figure 9:**
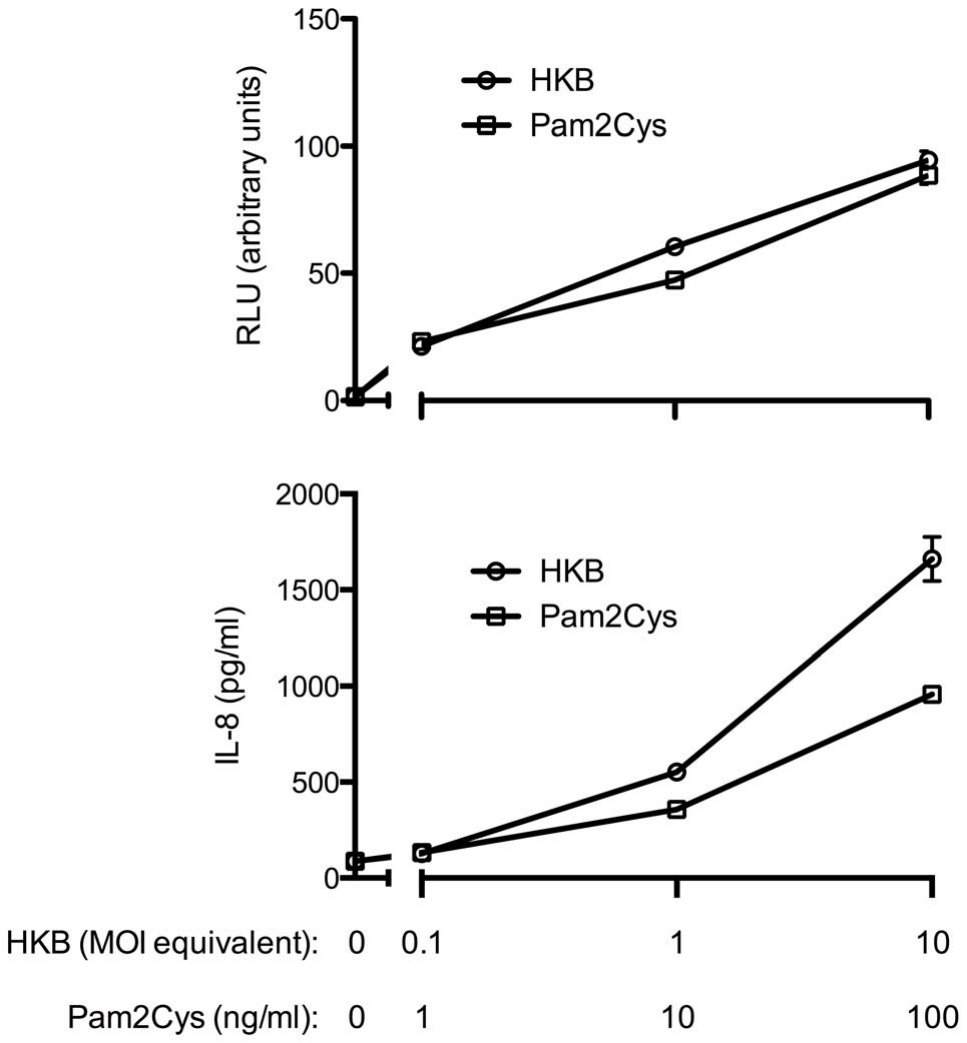
*B.* *abortus* strain S19 induces NF-κB activation in HEK293 cells overexpressing TLR2. HEK293 cells were transiently transfected with human TLR2. After stimulation with the diacyleted lipopeptide Pam2Cys or heat-inactivated *B. abortus* S19, NF-κB activation was assessed by a reporter gene assay (A), and IL-8 release by ELISA (B). Cells not transfected with TLR2 failed to exhibit NF-κB activation (not shown). Measurements were performed in triplicates, shown are mean values ± SD. One representative experiment out of two is shown.

When HEK293 cells were transfected with the LPS receptor consisting of TLR4 and MD-2, heat-inactivated S19 failed to induce a strong NF-κB activation as indicated by a reporter gene assay. Although at high concentrations faint NF-κB activation was observed, it was significantly weaker than LPS-mediated stimulation (Figure 8A). Furthermore, the cells completely failed to release IL-8 in response to *B. abortus* S19 further indicating that the bacteria are not recognized by TLR4 (Figure 8B). In contrast, both NF-κB activation and IL-8-release was induced by heat-inactivated bacteria in a dose-dependent manner in HEK293 cells overexpressing TLR2, which was comparable to the dose-dependent activation by a classical lipopeptide TLR2 ligand (Fig. 9). Additional transfection of TLR1 or TLR6 failed to further increase the response (data not shown). While this largely rules out an involvement of TLR1, the negative results obtained with the overexpression of TLR6 may be caused by the fact that HEK293 cells constitutively express TLR6 but not TLR1.

**Figure 10:**
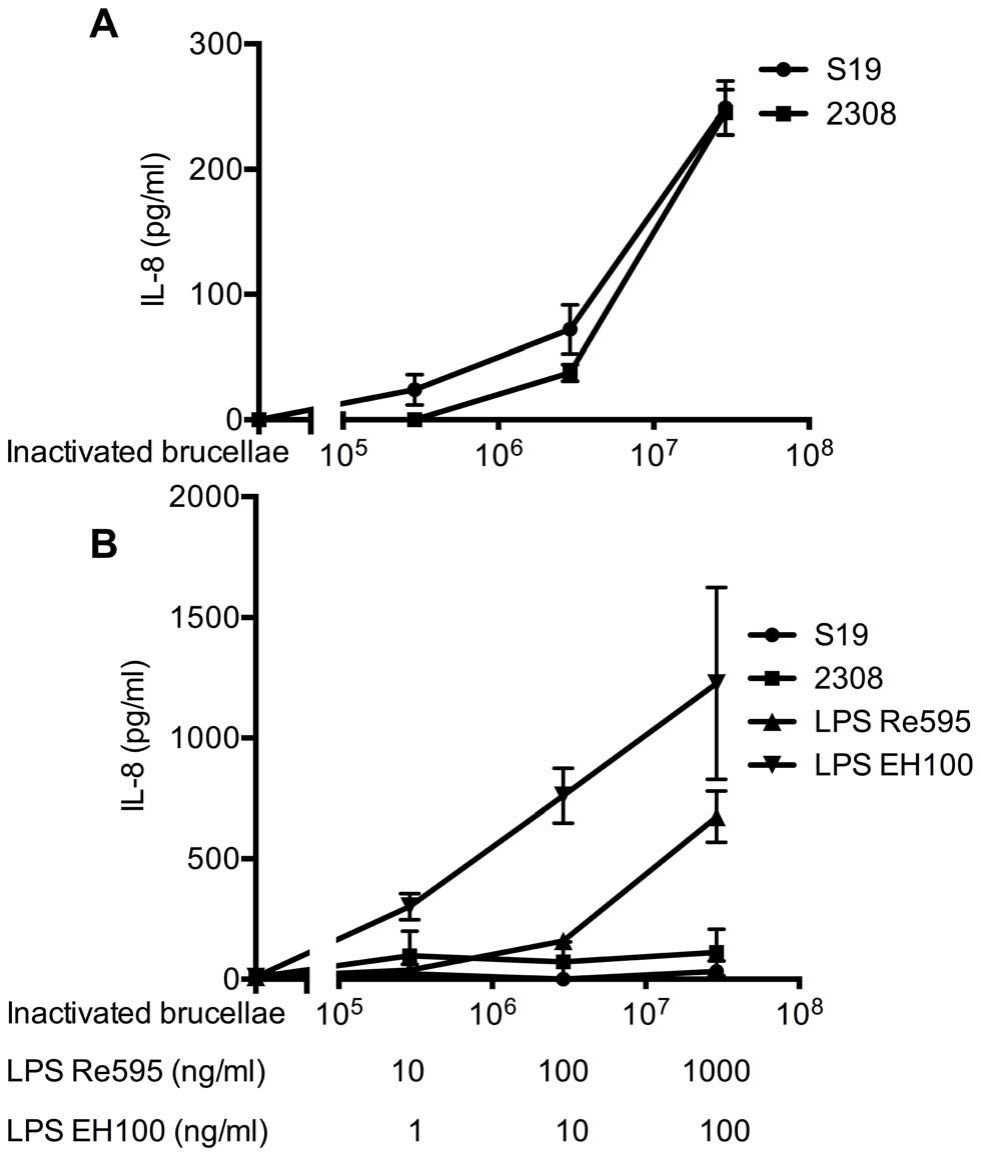
Both *B.* *abortus* S19 and strain 2308 induce IL-8 secretion by HEK293 cells overexpressing TLR2 but not TLR4. HEK293 cells were transiently transfected with human TLR2 (A) or TLR4 (B). After stimulation with LPS (rough, LPS Re595; smooth, LPS EH100) or heat-inactivated *B. abortus* S19 or strain 2308, NF-κB activation was assessed by IL-8 release by ELISA. Measurements were performed in triplicates, shown are mean values ± SD. One representative experiment out of two is shown.

Since these data for S19 were contradictory to those reported previously by Zwerdling et al. for heat-inactivated wild-type *B. abortus* 2308 [27], we compared these two *B. abortus* strains alongside in the same TLR overexpression system as before. We also included a smooth LPS (derived from *E. coli* EH100) as control stimulus to verify that our experimental system recognizes smooth LPS preparations. Notably, both *B. abortus* strains activated and induced IL-8 secretion in TLR2-expressing HEK293 cells in a comparable manner. In contrast, both *B. abortus* strains failed to induce IL-8 secretion in TLR4-expressing cells, which, however, were activated by two different LPS preparations (rough, LPS Re595; smooth, LPS EH100) and secreted IL-8 (Figure 10). Similar data were obtained in the reporter gene assay for intracellular NF-κB activation through *B. abortus* S19 and strain 2308 (data not shown). Thus, in the TLR overexpression system used, both *B. abortus* S19 and strain 2308 are primarily recognized through TLR2, most likely in form of the TLR2/6 heterodimer, but not TLR4. The lack of recognition by TLR4 cannot be explained by a general unresponsiveness of the transfected cells to TLR4 ligands since both LPS preparations, i.e., rough Salmonella LPS and smooth LPS derived from *E. coli*, were recognized by this system.

## Discussion

Microbial vectors are attractive for vaccine strategies aiming at the induction of strong cellular immune responses, and a large number of shuttles have been engineered from both viral and bacterial origin. The attenuated *B. abortus* S19 might comprise an advantageous bacterial vector since brucellae are much less pyrogenic than other enterobacteriae but has been shown to induce robust CD4^+^ and CD8^+^ T-cell responses in vivo [13], [15]. In the present study, we elucidated the interaction of *B. abortus* S19 with human DCs since their functions are crucial for the induction of immune responses against brucellae and thereby also against potential inserted foreign antigens.

First we investigated whether *B. abortus* S19 might hamper the maturation of human DCs. This has been shown previously for *B. suis* [29]. S19 altered the cytokine secretion profile in infected cells, i.e., infected DCs produced IL-12p70 while uninfected cytokine-matured DCs produced IL-12p40 but not IL-12p70, which is in agreement with previous observations [40]. In contrast to *B. suis*, *B. abortus* S19, did not interfere with DC-maturation although at later time points after infection the bacteria persisted in less mature cells. In contrast, S19 potently activated human DCs in the absence of other maturation stimuli. This was evident by enhanced surface expression of CD86, HLA-ABC, and HLA-DR, and a substantial increase in CD25, CD80, and CD83 expression, typical for mature DCs [26]. Activation of human DCs has also been observed for the virulent *B. abortus* strain 2308, which induced a comparable phenotype in monocyte-derived DCs as shown for S19 by us [27]. Since both DC-activating strains S19 and 2308 are smooth strains of *B. abortus*, the phenomenon of the bacterial interference with DC-maturation is not essentially associated with the presence of smooth LPS as suggested previously [41]. Zwerdling et al. [27] also observed secretion of IL-12p40 by 2308-activated DCs. Since p40 is a subunit shared by IL-12p70 and IL-23, we further characterized the cytokines and detected only IL-12p70 but not IL-23. Notably, infected DCs simultaneously produced the anti-inflammatory cytokine IL-10, independent of the presence of cytokines as further maturation stimulus. This simultaneous secretion of IL-10 and pro-inflammatory cytokines might control the duration and degree of the brucellae-induced inflammatory response.

Survival of brucellae for 48 h in human DCs has been documented before [25]. Here, we show that S19 is able to persist in DCs even for at least ten days without cytotoxic effects. Notably, the bacteria survived better in not fully mature than in mature cells. This was also true for DCs that received a maturation stimulus through cytokines. Future studies should address whether this survival also depends on the interaction of the virB type IV secretion system of the bacteria with the endoplasmic reticulum, as it has previously been shown for murine bone marrow-derived macrophages [42]. VirB proteins are expressed by S19 under acidic conditions, but less than by wild-type *B. abortus* at neutral pH [43].

The activating effect of S19 on DCs could not be transferred with supernatants from infected cells to uninfected DCs. Thus, cytokines, e.g., TNF-α that contributes to canarypox virus-induced maturation of human DCs [44], are not involved in the S19-induced DC-activation. Likewise, viable bacteria were not required for this process since heat-killed brucellae were equally able to induce DC-activation.

These results suggested that DC-activation induced by *B. abortus* S19 probably depends on heat-stable bacterial components recognized by pattern-recognition receptors, such as TLRs. We therefore investigated the involvement of TLR2 in combination with TLR1 or TLR6 and TLR4 in bacterial recognition and found that S19 activates cells primarily via TLR2 in combination with TLR6 but neither TLR4 nor TLR1. Similar data were obtained with heat-inactivated *B. abortus* 2308. Notably, TLR2 but not TLR4 engagement on DCs during immune priming is associated with a strong CD4^+^ memory T-cell response [45].

Possible bacterial ligands are the lipidated forms of Omp16 or Omp19, which in addition to heat-inactivated brucellae have been shown to induce TNF-α and IL-6 production by the human monocytic cell line, THP-1, in a TLR2-dependent manner [46]. TLR2-dependent IL-6 secretion is also involved in down-regulation of MHC-II molecules and of the antigen processing capacity of THP-1 cells infected with wild-type *B. abortus* [47]. Also in the murine system, *B. abortus*-induced secretion of IL-12p40 by bone marrow-derived DCs depended on TLR2 [37]. Notably, another study revealed that *B. abortus*-induced secretion of IL-12p40 in murine splenic DCs was independent of TLR2 [48], and TLR2 knockout mice were able to control *B. abortus* infection similar as wild-type animals indicating that pathogen-recognition through TLR2 is not essential for resistance to *B. abortus* in the murine brucellosis model [49], [50]. Our data regarding the involvement of TLR6 (together with TLR2) in the cellular recognition of *B. abortus* is supported by a recent study by de Almeida et al. [51] who also showed that TLR6 cooperates with TLR2 in sensing *B. abortus* strain 2308. In addition, the authors reported that TLR6 (but not TLR2) is required for the in vivo control of the bacteria in the murine model of brucellosis, and that murine DCs lacking TLR2 or TLR6 are unable to produce of TNF-α or IL-12 [51].

In contrast, Zwerdling et al. [27] showed that cytokine production of human DCs incubated with heat-killed wild-type strain 2308 depends on both TLR2 and TLR4. Similarly, both receptors are involved in S19-induced TNF-α production by murine macrophages but dispensable for the clearance of strain S19 following intraperitoneal infection of mice [50]. In contrast, TLR4 knockout mice showed significantly higher bacterial burden than control animals after infection with strain 2308 [49], and the *Brucella* lumazine synthase [52], or the protein moiety of Omp16 [53], or both, rather than Brucella LPS, are putative ligands in TLR4-mediated protection against *B. abortus*. These inconsistent results may be due to distinct experimental approaches, particularly, the different cell types used as reporter systems.

DCs incubated with *B. abortus* S19 secreted substantial amounts of IL-12p70 although the opposite is characteristic for cells stimulated only through TLR2 [54]. Thus, the involvement of an additional receptor in DC-activation is likely, and synergistic effects of, e.g., distinct TLR ligands on IL-12 production have been reported [55].

Where as other TLRs expressed by human DCs, such as TLR5 or TLR3 and TLR8 are unlikely involved in DC-activation by brucellae, which lack flagella, dsRNA, and ssRNA, other innate receptors, such as the cytosolic nucleotide oligomerization domain (NOD) receptors (*B. abortus* localizes intracellularly) might contribute to this process. Although Oliveira et al. [56] have recently shown that NOD1 or NOD2 knockout mice were equally susceptible to infection with the S2308 strain as wild-type animals, this does not exclude the contribution of one of these receptors in DC-activation since similar data have been reported for mycobacterial infections. In experimental tuberculosis, NOD knockout mice were similarly susceptible to *M. tuberculosis* infection while NOD-deficient DCs produced less IL-12 as well as IL-6 and IL-10 upon stimulation with mycolylarabinogalactan peptidoglycan [57]. Whether non-leukocytes, e.g., endothelial cells that produce pro-inflammatory cytokines in response to the infection with *B. abortus* in vitro [58], contribute to the induction of specific immune responses *in vivo* needs to be determined. Other receptors possibly involved in S19-induced DC-activation comprise cytosolic receptors recognizing bacterial nucleic acids, e.g., those of the Rig-I like receptor (RLR) family, or other yet undefined DNA sensor receptors [35].

In conclusion, *B. abortus* S19 infects and activates human DCs without causing apparent cytotoxicity, and TLR2 is involved in the recognition of the bacteria. Since the genome of S19 has recently been analyzed [59], the inactivation of genes encoding defined virulence factors will yield further attenuated strains on the background of *B. abortus* S19. Our data encourage future work, e.g., in vivo studies in non-human primates, regarding the use of such bacteria as vaccine vectors for the induction of Th1 immune responses.

## Supporting Information

Figure S1 **Expression of DC maturation markers by DCs upon infection with** ***B. abortus*** **S19 and cultured in the presence of pro-inflammatory cytokines.** Monocyte-derived immature DCs were infected with *B. abortus* S19 (MOI, 20) for 1 h, the bacteria were washed out, and the cells incubated in the presence of pro-inflammatory cytokines (TNF-α, IL-1β, IL-6, PGE_2_). Uninfected control cells were incubated alongside in the presence or absence of cytokines. After 48 h, the phenotype of the cells was determined by flow cytometry. Medians of the MFIs as well as the 25% and 75% percentiles of the MFIs of six independent experiments (MFIs of isotype controls were subtracted). * p<0.05 compared to untreated DCs (Wilcoxon matched-pairs signed rank test).
(TIFF)

Figure S2 **Expression of DC maturation markers by** ***B. abortus*** **S19-infected DCs.** Monocyte-derived immature DCs were infected with *B. abortus* S19 (MOI, 20) for 1 h, the bacteria were washed out, and the cells were incubated for another 48 h. Control cells were left uninfected. After 48 h, the phenotype of the cells was determined by flow cytometry. Medians of the MFIs as well as the 25% and 75% percentiles of the MFIs of six independent experiments (MFIs of isotype controls were subtracted). * p<0.05 compared to untreated DCs (Wilcoxon matched-pairs signed rank test).
(TIFF)

Figure S3 **Expression of DC maturation markers by DCs incubated with heat-inactivated** ***B. abortus strain*** **S19.** Immature DCs were incubated in the presence of heat-inactivated *B. abortus* S19 (equivalent to MOI 10) or kept in GM-CSF and IL-4 as immature cells. After 48 h, the phenotype of the cells was characterized by flow cytometry. Medians of the MFIs as well as the 25% and 75% percentiles of the MFIs of six independent experiments (MFIs of isotype controls were subtracted). * p<0.05 compared to untreated DCs (Wilcoxon matched-pairs signed rank test).
(TIFF)
