# Supplementary figures and images for: The Attenuated Brucella abortus Strain 19 Invades, Persists in, and Activates Human Dendritic Cells, and Induces the Secretion of IL-12p70 but Not IL-23
Source: PLoS One. 2013 Jun 21;8(6):e65934. doi: 10.1371/journal.pone.0065934 (PMC3689767; doi:10.1371/journal.pone.0065934)

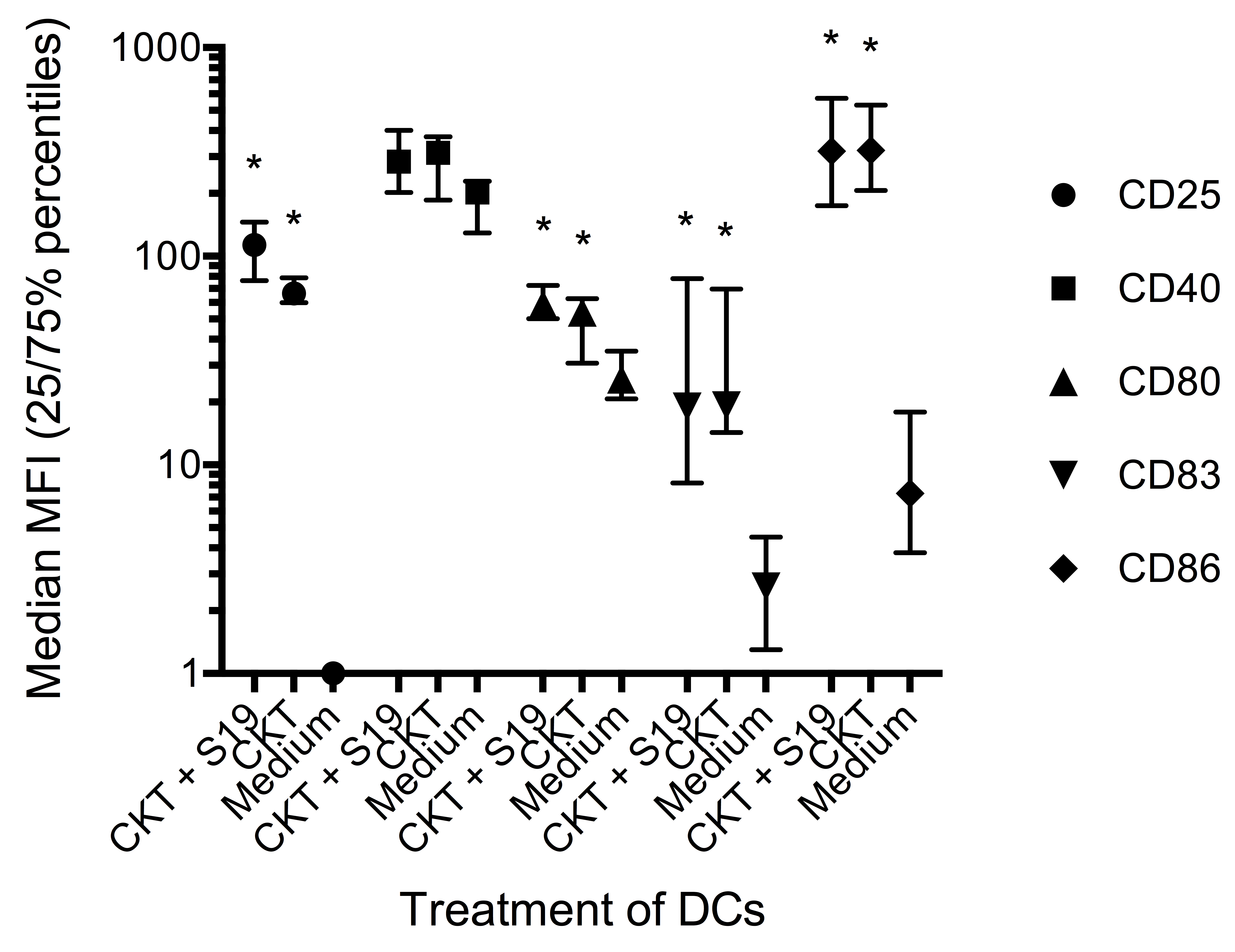

Supplement: Figure S1 — Expression of DC maturation markers by DCs upon infection with B. abortus S19 and cultured in the presence of pro-inflammatory cytokines. Monocyte-derived immature DCs were infected with B. abortus S19 (MOI, 20) for 1 h, the bacteria were washed out, and the cells incubated in the presence of pro-inflammatory cytokines (TNF-α, IL-1β, IL-6, PGE2). Uninfected control cells were incubated alongside in the presence or absence of cytokines. After 48 h, the phenotype of the cells was determined by flow cytometry. Medians of the MFIs as well as the 25% and 75% percentiles of the MFIs of six independent experiments (MFIs of isotype controls were subtracted). * p<0.05 compared to untreated DCs (Wilcoxon matched-pairs signed rank test). (TIFF) [file pone.0065934.s001.tiff]

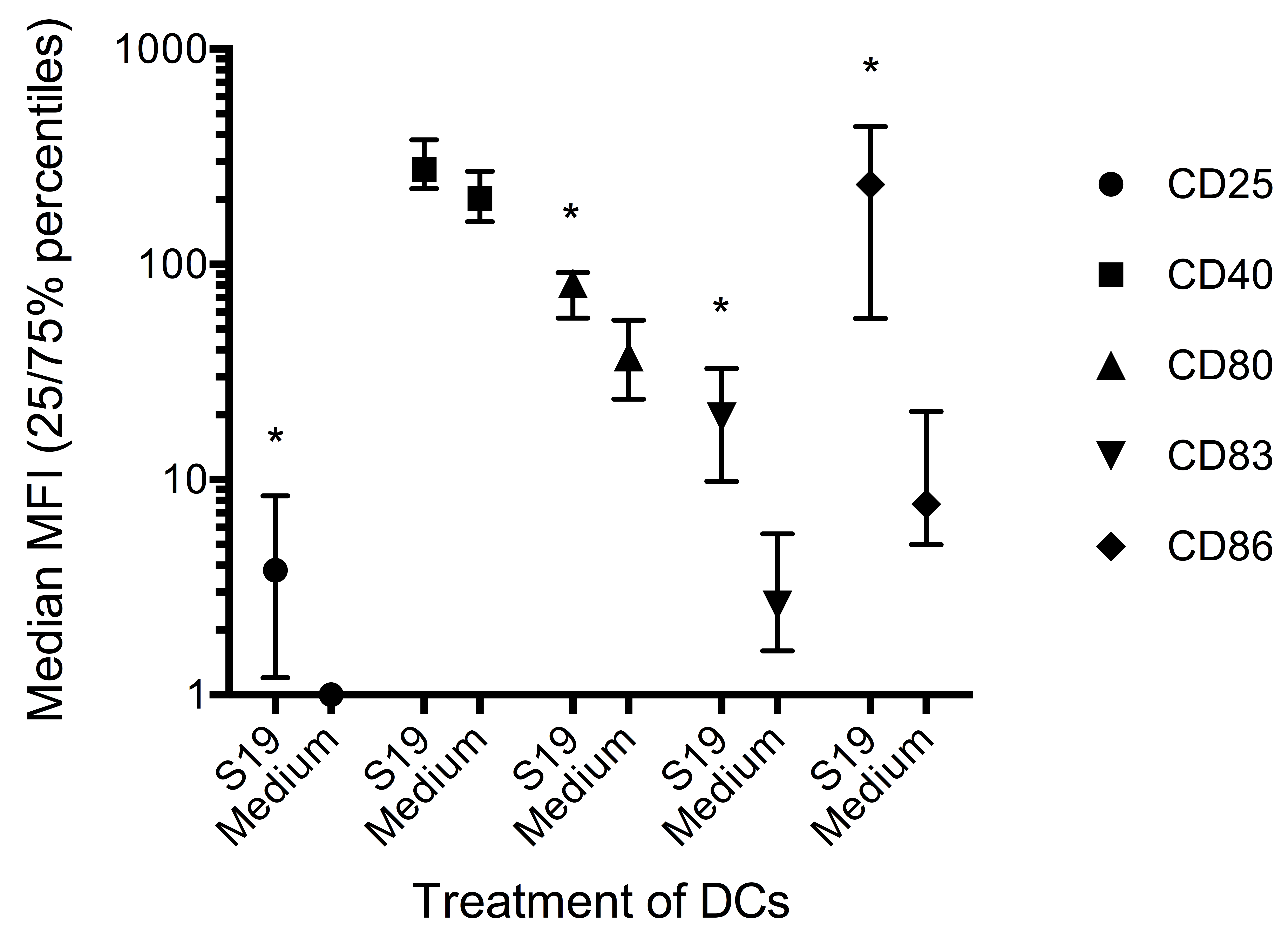

Supplement: Figure S2 — Expression of DC maturation markers by B. abortus S19-infected DCs. Monocyte-derived immature DCs were infected with B. abortus S19 (MOI, 20) for 1 h, the bacteria were washed out, and the cells were incubated for another 48 h. Control cells were left uninfected. After 48 h, the phenotype of the cells was determined by flow cytometry. Medians of the MFIs as well as the 25% and 75% percentiles of the MFIs of six independent experiments (MFIs of isotype controls were subtracted). * p<0.05 compared to untreated DCs (Wilcoxon matched-pairs signed rank test). (TIFF) [file pone.0065934.s002.tiff]

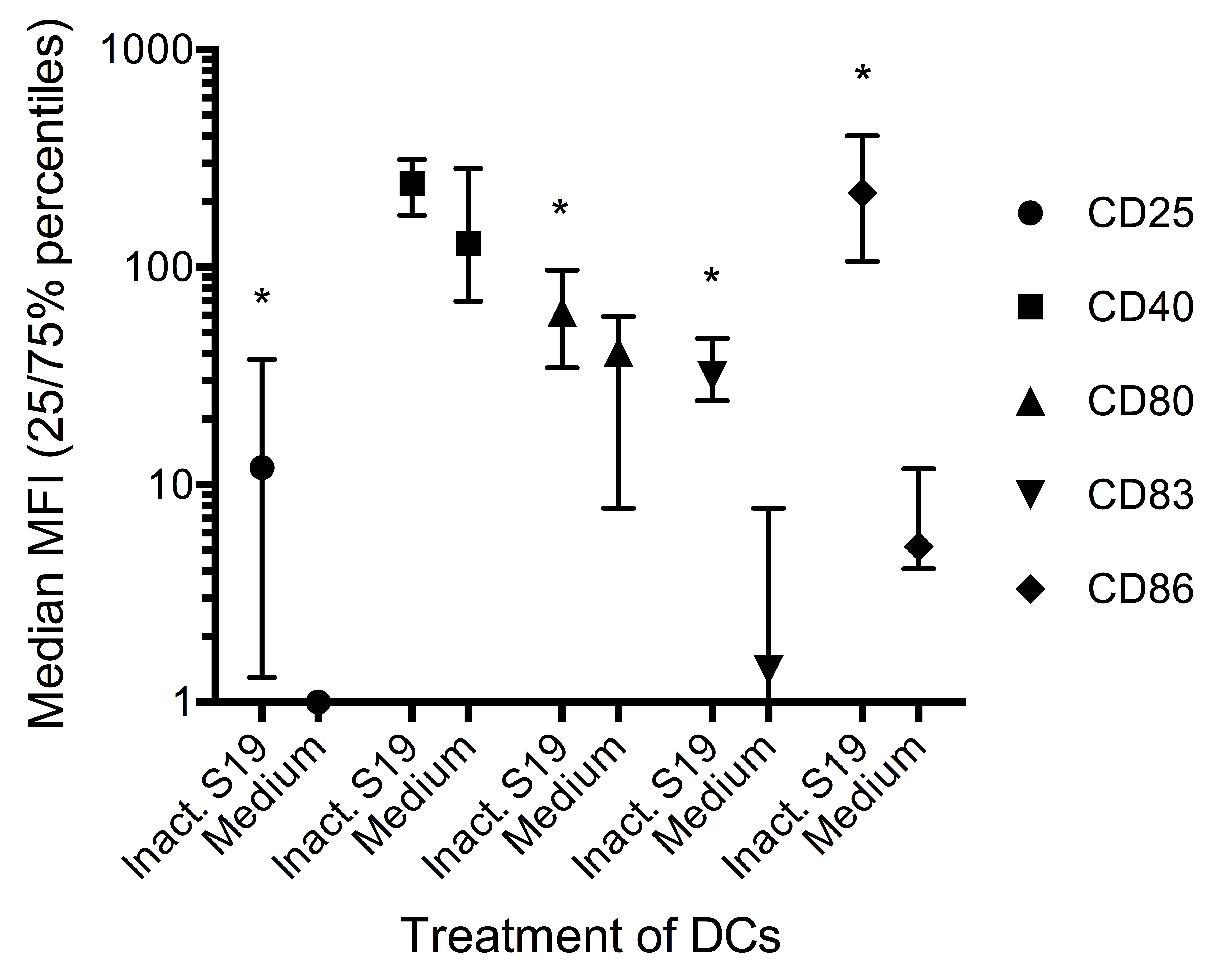

Supplement: Figure S3 — Expression of DC maturation markers by DCs incubated with heat-inactivated B. abortus strain S19. Immature DCs were incubated in the presence of heat-inactivated B. abortus S19 (equivalent to MOI 10) or kept in GM-CSF and IL-4 as immature cells. After 48 h, the phenotype of the cells was characterized by flow cytometry. Medians of the MFIs as well as the 25% and 75% percentiles of the MFIs of six independent experiments (MFIs of isotype controls were subtracted). * p<0.05 compared to untreated DCs (Wilcoxon matched-pairs signed rank test). (TIFF) [file pone.0065934.s003.tiff]
